# Quasi mode-locking of coherent feedback random fiber laser

**DOI:** 10.1038/srep39703

**Published:** 2016-12-22

**Authors:** R. Ma, W. L. Zhang, X. P. Zeng, Z. J. Yang, Y. J. Rao, B. C. Yao, C. B. Yu, Y. Wu, S. F. Yu

**Affiliations:** 1 Key Laboratory of Optical Fiber Sensing and Communications (Education Ministry of China), University of Electronic Science and Technology of China, Chengdu 610054, China; 2 Department of Applied Physics, Hong Kong Polytechnic University, Hung Hom, Hong Kong SAR, China

## Abstract

Mode-locking is a milestone in the history of lasers that allows the generation of short light pulses and stabilization of lasers. This phenomenon is known to occur only in standard ordered lasers for long time and until recently it is found that it also occurs in disordered random lasers formed by nanoscale particles. Here, we report the realization of a so-called quasi mode-locking of coherent feedback random fiber laser which consists of a partially disordered linear cavity formed between a point reflector and a random distributed fiber Bragg grating array with an inserted graphene saturable absorber. We show that multi-groups of regular light pulses/sub-pulses with different repetition frequencies are generated within the quasi mode-locking regime through the so-called collective resonances phenomenon in such a random fiber laser. This work may provide a platform to study mode locking as well as pulse dynamic regulation of random lasing emission of coherent feedback disordered structures and pave the way to the development of novel multi-frequency pulse fiber lasers with potentially wide frequency tuning range.

Disorder exists widely in various photonic structures, carrying a large amount of interesting physics^1^,^2^. Commonly, suppression of optical random feedback in such structures is necessary in order to reduce the undesired detrimental effect such as scattering loss and dephasing. While in some cases, disorder can be used to realize unconventional optical devices with special performance such as incoherent lasers, high efficiency photovoltaics and sensors^3^,^4^,^5^. In the past few years, random fiber lasers (RFLs) have experienced extensive investigations. Thanks to the unique characteristics, such as simple structure and fabrication, low noise, low coherence, potentially high power/efficiency, RFLs have shown their promising potentials as a new generation of light sources for telecommunication, sensing, and imaging applications^6^,^7^,^8^. Since the realization of random distributed feedback fiber lasers in 2010, various types of incoherent feedback RFLs have been proposed and demonstrated^9^,^10^,^11^,^12^, wherein pulse RFLs are of great significance for both scientific values and potential applications^13^,^14^,^15^,^16^. However, due to the use of incoherent feedback, this type of RFLs exhibit “modeless” characteristics which block the realization of mode-locking as if in conventional fiber lasers with ring or Fabry-Perot cavities^17^,^18^,^19^,^20^.

Mode locking, which has shown great potential in applications of laser kinetics and ultrafast optics^21^,^22^, is known to occur only in standard ordered lasers for long time and until recently it is found that it also occurs in disordered random lasers formed by nanoscale particles^23^,^24^,^25^. Furthermore, localized feedbacks can also be used as effective reflectors to realize resonant random lasing^26^,^27^. However, mode locking of RFLs, especially, ordered light pulse generation through mode locking of RFLs is still a problem to be solved. One promising way to solve this problem is to use coherent feedback RFLs (CFRFLs)^28^,^29^,^30^, e.g., RFL made from active fibers and inscribed with random distributed fiber Bragg grating (RD-FBG) arrays, because light localization and coherent laser modes are relatively easy to be obtained in such disordered structures. Besides, unique resonance channels and numerous cavity modes of CFRFLs would provide a prospective way for mode locking and dynamical regulation of laser outputs^30^, giving birth to a brand new type of pulsed RFLs. For example, CFRFLs with various lasing modes have the potential of achieving multi-frequency generation or wide tunable frequency range in the mode-locking regime, which is difficult to be realized in conventional mode-locked lasers with fixed cavity lengths^17^,^18^,^19^,^20^.

Here we propose and demonstrate a so-called quasi mode-locking of CFRFLs, which is formed between a point reflector and a RD-FBG array recorded in Erbium-doped fiber (EDF) with an incorporated graphene saturable absorber (GSA). We obtain two regimes of resonant modes in such a CFRFL with partially disordered resonances, corresponding to the global (low frequency regime) and the localized (high frequency regime) cavities, respectively. Hence, a unique phenomenon, called collective resonances between modes of the two regimes, is observed. Through the assistance of saturable absorption of the GSA, resonant modes can be selected and mode-locked in the quasi Q-switched mode-locking regime. As a result, regular multi-group light pulses/sub-pulses with varying repetition rates can be generated by the proposed CFRFL. These novel features of quasi mode locking of CFRFLs with a partially disordered cavity make it possible to generate or tune regular laser pulses at different repetition rates more effectively than a conventional fiber laser with a fixed cavity length. The study could also be a useful attempt for dynamic regulation of light behavior in disordered structures.

## Results

### Experimental method

**Figure 1:**
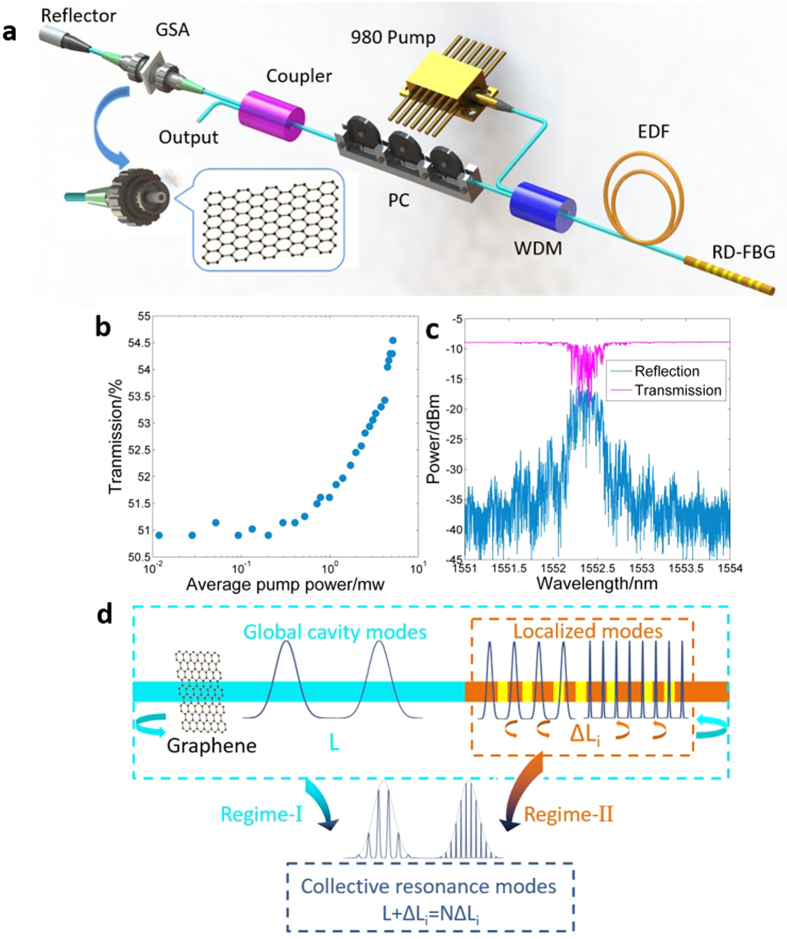
Schematic of quasi mode-locking of coherent feedback Q-switched random fiber laser and diagram of the laser cavity. (**a)** Schematic of the Q-switched mode-locking random fiber laser. Reflector: fiber reflector with average reflectivity ≥97.5%, GSA: graphene saturable absorber, Coupler: 1:99 coupler with 1% port as output, PC: polarization controller, WDM: wavelength division multiplexer, EDF: Erbium doped fiber, RD-FBG: random distributed FBG array, inset: detail schematic of graphene based saturable absorber. (**b)** Nonlinear transmission of GSA. (**c)** Reflection and transmission spectra of the RD-FBG. (**d)** Schematic diagram of the laser cavity. L represents the geometric length between fiber reflector and the RD-FBG, Δ*L*_*i*_ represents the random optical path length within the RD-FBG due to multi-reflection. The dashed rectangles with different colors reveal the relationship of mode oscillations for Regime-I and Regime-II.

The proposed CFRFL has a linear cavity as shown by Fig. 1a (see Methods for details). The GSA was made by covering a fiber pigtail facet with graphene through optically driven deposition method (see Methods for details). We measured the nonlinear absorption of graphene through the conventional balanced twin-detector measurement system. As is shown in Fig. 1b, the transmission boosts from 51% to 54.5% with input pump power increasing from 0.01 to 10 mW, which is induced by Pauli blocking effect. Hence optical modulation depth of the GSA is 3.5%. The RD-FBG array consists of 20 FBGs with the same central wavelength (see Methods for details). Figure 1c shows the reflection/transmission spectrum of the FBG array, wherein several spikes/dips are observed, indicating coherent interferences of random feedback in the RD-FBG. The localization length of the FBG array *ξ* is calculated from *T*(*L*) ≈ exp(−0.5L/*ξ*) to be 3.7 cm, wherein L (=15.5 cm) is the length of FBG array and T (~0.126) is the average transmission. Obviously, the localization length is shorter than the length of FBG array, satisfying the requirement of light localization which is essential for coherent RFLs^31^, thus CFRFL can be obtain by using this RD-FBG array as a random reflector.

### Operational principle

In the proposed structure, there are several cavity modes related to two oscillation regimes of light. The first one corresponds to global cavity modes formed between the point reflector and the RD-FBG (i.e., low frequency regime, Regime-I), and the second is localized modes induced by multiple reflection and interference within the RD-FBG itself (i.e., high frequency regime, Regime-II).

The resonances are shown in Fig. 1d. *L* is the geometric length between the reflector and the RD-FBG array, and Δ*L*_*i*_ corresponds to different light path length of multiple reflection within the RD-FBG. For Regimes I and II, the cavity length can be expressed as *L* + Δ*L*_*i*_ and Δ*L*_*j*_, respectively. Thus, Regime-I supports oscillation (global cavity) modes of several values of cavity length, which might generate mode-locking pulses at different repetition rates. Additionally, oscillation modes of Regime-II may resonate with high-order harmonic modes of Regime-I (i.e., *L* + Δ*L*_*i*_ = *N*Δ*L*_*j*_, *N* is an integer), giving birth to sub-pulses at higher repetition rates. Through combination of saturable absorption and collective mode resonance mentioned above, a few of these modes could be selected and mode locked. This provides an efficient and flexible way to vary and multiply repetition rate of light pulse through mode locking.

### Experimental results

**Figure 2:**
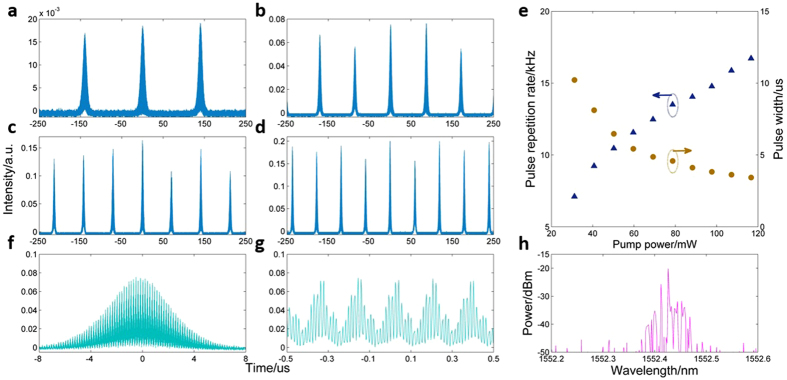
Temporal-spectra characteristics of coherent feedback Q-switched random mode-locking laser. (**a–d)** Oscilloscope traces of the Q-switched trains under pump power of 31.15, 59.61, 88.23 and 116.67 mW, respectively. (**e)** Pulse repetition rate and pulse width as a function of pump power. (**f)** A single Q-switched pulse under pump power of 59.61 mW (close-up view of **b**). (**g**) Mode locking pulses/sub-pulses within the Q-switched pulse (close-up view of **f**). (**h)** The optical spectrum under pump power of 59.61 mW.

When pump power was increased above 21.15 mW, passive Q-switched pulses was achieved. Figure 2a–d plot the pulse sequence of the Q-switched regime versus different pump power. It is obvious that pulse repetition rate increases with the pump power from 7.1 to 16.7 kHz while temporal width of the Q-switched pulse envelop decreases from 10.2 to 3.4 μs, which follow typical feature of passive Q-switching operation^32^, seeing Fig. 2e. To further verify that the passive Q-switching was attributed to GSA, we replaced the graphene-coated fiber pigtail with a common clean one. Under this circumstance, no Q-switching operation was observed even the pump power is high enough.

Close-up view of one single Q-switched event under typical pump power of 59.61 mW is shown in Fig. 2f, wherein mode-locking pulses can be clearly observed within the Q-switched envelope. Unlike the typical Q-switched mode-locking pulse^33^, we observed mode-locking pulses in both the low frequency and the high frequency regimes. For example, low frequency pulses (see Fig. 2f) of each round trip of the global cavities also include multiple sub-pulses (see Fig. 2g) that correspond to specific localized oscillation modes in the RD-FBG. The full width at half maximum of the sub-pulses is estimated to be 7 ns in Fig. 2g. The optical spectrum of the Q-switched pulses under pump power of 59.61 mW is given by Fig. 2h. We can see that the power distribution in spectra domain exhibit fluctuations with different wavelength separation that correspond to multimode oscillation and coherent interference of multiple reflections of the RD-FBG. In ref. 24, mode locking in a nanoparticle based random laser is achieved through strong interacting of individual localized modes by modulating the pumping region, which is identified by spectra evolution from narrow spikes into a relatively broad and smooth one. Here a different way of mode locking under assistance of GSA is applied. To reveal the complex dynamics of the proposed laser, waveforms in the time domain and RF spectrum (other than spectrum) of the output are mainly discussed.

**Figure 3:**
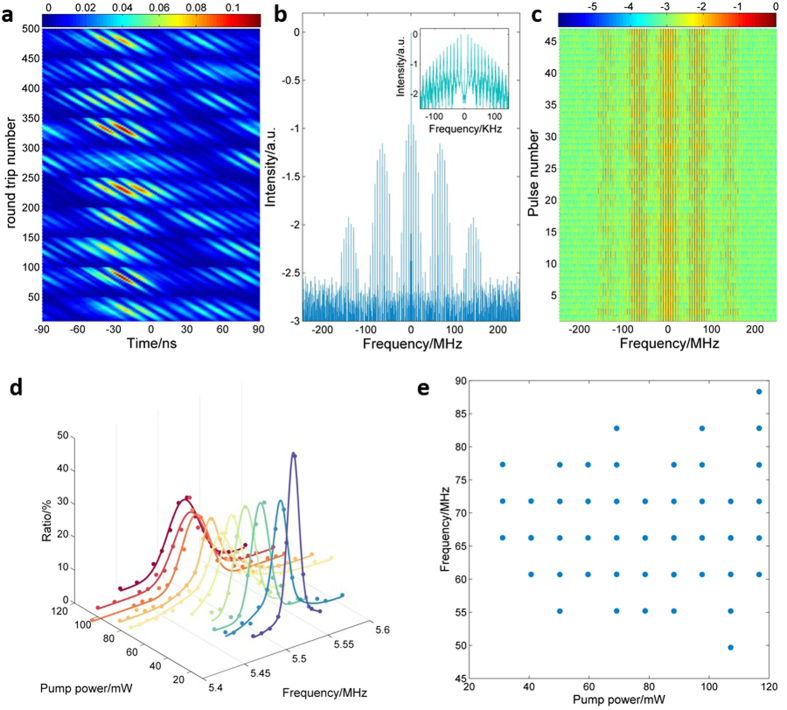
Dynamical spatio-temporal and frequency characteristics of the coherent feedback Q-switched laser correspond to pump power of 59.61 mW and statistical analysis of the frequency components versus pump power. (**a**) Spatio-temporal intensity dynamics of mode-locking regime for cascaded Q-switched events for pump power of 59.61 mW. (**b**) Radio frequency spectrum of the pulse series for time scale of 4 ms for pump power of 59.61 mW. (**c**) Map of the radio frequency spectrum evolution for cascaded Q-switched events for pump power of 59.61 mW. (**d**) Ratio of low frequency components appeared in mode-locking regime as a function of pump power. (**e**) Summary of all the high frequency components at different pump power.

To get a better understanding of the mode-locking pulse evolution, spatio-temporal intensity dynamics under pump power of 59.21 mW is given in Fig. 3a, where cascaded intensity dynamics of each low-frequency round trip period (i.e., successive time series with equal periods of 182 ns, as presented in Fig. 2g) are selected apart and arranged row-wise (see Supplementary Figure S1 for details, the methods for obtaining Fig. 3a also see Supplementary I for details)^34^. To make the evolution within each Q-switched pulse more noticeable, the long range of time separation between neighboring Q-switched pulse is removed in time domain. In the vertical direction of Fig. 3a, bright and dark regions appear alternatively, corresponding to the consecutive Q-switched events, while in the horizontal direction, several bright stripes indicating mode locking evolution of the sub-pulses are observed. It is also clear that the intensity dynamics of the pulses within the same Q-switched period remain consistent (i.e., evidence of mode locking), but the temporal profiles of different Q-switched events are different from each other.

Figure 3b gives the radio frequency (RF) spectrum of the pulse series through fast Fourier transform (FFT) analysis for a total time scale of 4 ms under pump power of 59.61 mW. In the spectrum, frequency spikes appear at a beating frequency of ~5.5 MHz, reflecting the fundamental repetition frequency of the global-cavity related to low frequency regime (Regime-I). Besides, the total spectrum exhibits a distinct multi-peak envelop with peak separation around 70 MHz, which corresponds to the localized oscillation frequency of the RD-FBG (Regime-II). Coexistence of the two frequency components reflects resonant oscillation of individual modes between the low and the high frequency regimes, which generates both the mode-locking pulses and sub-pulses, and it is a distinct character compared with conventional Q-switched mode-locking fiber laser. The inset Figure shows the enlarged detail of the near zero frequency spikes, wherein beating frequency of about 11.87 KHz are observed, coinciding with the Q-switched repetition rate. Figure 3c illustrates the map of RF spectrum evolution for cascaded single Q-switched period. It is observed that the shapes of the spectra are similar for different Q-switched periods, except slightly randomness in peak amplitude and beating frequency. To verify the function of the RD-FBG array, RF spectrum of a conventional Fabry-Perot cavity formed by replacing the RD-FBG with a single high reflectivity FBG has been discussed and no high frequency regime exists (see Supplementary Figures S2 and S3 for details).

Random structure of the RD-FBG introduces multiple fundamental frequencies in both Regimes I and II. For Regime I, there exists several beating frequencies. A statistical analysis of the beating frequencies, i.e., probability distribution of the low frequencies, of the mode-locking pulses/sub-pulses within one single Q-switched period is shown in Fig. 3d, taking 20 cascaded Q-switched periods as the sample for each pump power. It is observed that most of the low frequency components concentrate in the range from 5.50 to 5.56 MHz, and the distribution of the frequency components become broader with increasing of the pump power. It is presumed that with pump power increasing, more modes within bandwidth of the random distributed FBG array with lower Q-factor can be excited and mode locked, which directly leads to the frequency spectrum broadening. It is interesting to note that the frequency components between 5.4 and 5.6 MHz (as shown in Fig. 3d) correspond to variation of effective cavity length [*f* = *c*/2*nL*, wherein c (~3 · 10^8^ m/s) and n (~1.468) are speed of light wave in vacuum and the refractive index of the fiber, respectively, L is the effective cavity length] from 18.92 to 18.38 m. The difference of effective cavity length can be as large as 0.54 m, which is much longer than the geometrical length of the RD-FBG, indicating that the optical path length of light could be extended due to multiple reflections within the RD-FBG. Other factors, such as effective refractive index change, cavity length elongation due to thermal expansion, and even the effective reflection center change of the RD-FBG array couldn’t lead to so much difference in cavity length. This is a significant feature compared with mode-locking lasers using a fixed cavity length.

The appearance of Regime-II that is related with the localized resonance, is another peculiarity of the proposed laser. Using the same sample of the 20 cascaded Q-switched periods, Fig. 3e plots all the beating frequencies of Regime-II under different values of pump power. It is a statistic analysis of the RF spectra (that obtained through independent FFT analysis of the waveforms of the 20 Q-switched pulses under each pump power), and we only record the frequency components related with the high frequency regime. It is observed that the beating frequencies concentrate in the region between 50 and 90 MHz. Besides, the high beating frequencies in Regime-II well coincide with the high-order harmonic of the low beating frequency 5.52 MHz in Regime-I, reflecting the collective oscillation effect between regimes I and II. This is because that the RD-FBG induces light oscillations of various frequencies, and those coincide with high order harmonics of the low beating frequency will be selected during mode competitions, while other random modes can be suppressed. Thus, mode-locked sub-pulses at higher repetition rate can be obtained. It is worth noting that, in conventional mode-locking lasers, high-order harmonics of the fundamental mode can be generated through nonlinear effects^35^, but the order of the harmonics depends on the value of pump power. In our study, the case is different, i.e., the range of the high frequency components is insensitive to the pump power, reflecting that the RD-FBG plays a dominant role for the sub-pulse generation.

### Numerical analysis

**Figure 4:**
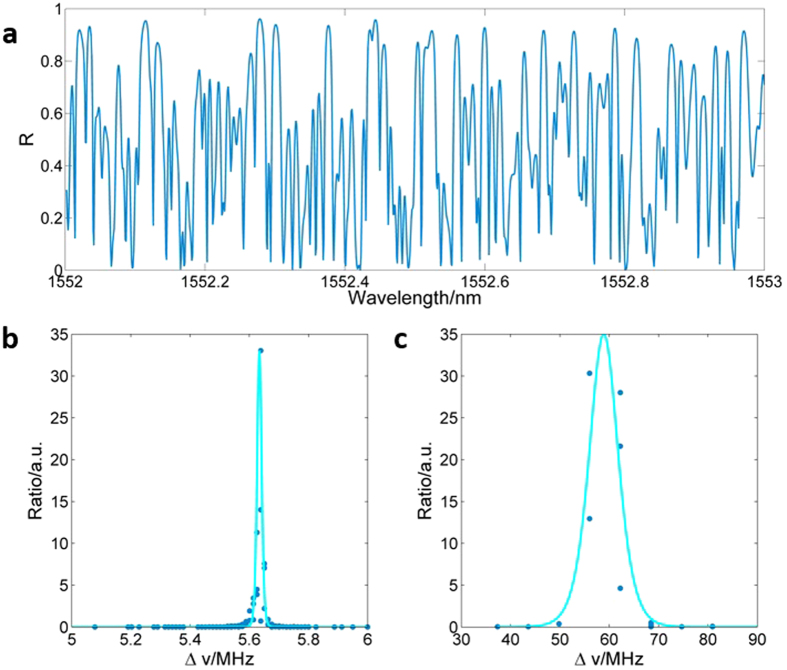
Simulated reflection spectrum and frequency distribution for global cavity modes and localized modes of the RD-FBG structure. (**a**) Reflection spectrum. (**b**) Ratio distribution of frequency for global cavity modes with fitting curve (simulation resolution of frequency is 0.01 MHz). (**c**) Ratio distribution of frequency for localized modes with fitting curve (simulation resolution of frequency is 5 MHz).

The RD-FBG based cavity presents complex resonances comparing to conventional single Fabry-Perot cavity. Using transfer matrix method^36^, characteristics of the frequency for both global cavity modes and localized modes can be revealed (see Methods for details). Figure 4a shows the reflection spectra of the RD-FBG with simulating resolution of 1 pm, which is much higher than that of the OSA (Ando, spectral resolution is 0.01 nm) used in the experimental section. Thus, by setting the simulating resolution into specific values, detailed resonant mode characteristics can be revealed through cold cavity analysis of the simulated reflection spectrum.

Resonant information of the considered structure is obtained by statistical analysis of frequency separations, Δ*υ* (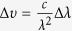, *υ* is frequency, *c* is speed of light wave in vacuum, λ is light wavelength, and Δ*λ* is wavelength separation), of neighboring peaks in the reflection spectrum. The ratio distribution of Δ*υ* for global and localized cavity modes are given in Fig. 4b and c, respectively. It is worth specially emphasizing that the simulating resolutions of Fig. 4b and c are set to be 0.01 and 5 MHz respectively to reveal the frequency characteristics in Regime I and II. For regime I, the majority of the global cavity modes concentrate within the frequency range between 5.58 and 5.65 MHz. For regime II, frequency components are mainly observed between 40 and 90 MHz, due to collective oscillation between the global and the localized cavity modes. All the theoretical analysis results coincide well with the experimental ones, which further verifies that regimes I and II derive from coherent interferences of light in the partially disordered laser cavity, and collective resonances of the two regimes determine the ranges of their frequency components.

## Discussion

The above results clearly show that quasi mode-locking and regular pulses can be obtained in a coherent feedback disordered structure. The proposed laser cavity with a RD-FBG array enables existence and interaction of resonant regimes for both the global and localized modes. Several laser modes of these two regimes can be mode locked through the assistance of the GSA, and locked modes of the two regimes also show collective resonances. These effects enable pulse generation of multiplied and varied frequencies without changing the laser cavity length. By correctly designing the parameters (e.g., number, separation and reflectivity) of the RD-FBG, it is promising to generate mode-locked pulses with more/higher frequencies over a wider spectral range. The universal nature of our all-fiber version demonstrated could also be grafted onto other kind of laser cavities, such as active on-chip lasers, providing platforms for investigating or controlling mode and dynamical characteristics of complex cavities.

In conclusion, a passively Q-switched quasi mode-locked CFRFL has been realized. Quasi mode-locked pulses and sub-pulses with multiple repetition frequencies were obtained within different Q-switched events. The mode-locked pulses correspond to several cavity lengths due to the presence of the RD-FBG. The combination of RD-FBG and GSA provides a novel mechanism of generating collective resonances over the low and high frequency regimes, thus, sub-pulses with higher repetition rates (tens of the low beating frequency) can be realized. Further statistical analysis indicates that the mode-locking of pulses/sub-pulses have a few repetition rates in different Q-switched envelops, which is a unique feature of the proposed pulse CFRFL. This work demonstrates the possibility of mode locking as well as pulse dynamic regulation of random lasing emission in a coherent feedback disordered structure with the assistance of resonant mode interaction and gain saturation. It also provides an effective and flexible method to vary repetition rates of mode-locked pulses at much larger extent compared to conventional pulsed fiber lasers with fixed cavity length. It is likely to pave a way for dynamic manipulation and regulation of output modes in coherent feedback disordered photonic systems.

## Methods

### Experimental setup

A fiber reflector and the RD-FBG array form the linear cavity of about 18.08 m. A 980 nm pump is coupled into a 6 meter long Erbium doped fiber (EDFC-980-HP, Nufern) through 980/1550 nm WDM. The 1550 nm port of the WDM is connected with a polarization controller (PC). The 1% port of a 1:99 coupler is used for monitoring the laser output by optical spectrum analyzer (OSA, Ando, spectral resolution is 0.01 nm), photodetector (Conquer, 10 GHz bandwidth), oscilloscope (Rohde & Schwarz, 1 GHz bandwidth and 5 GSa/s sampling rate) and power meter. The GSA is inserted between the fiber reflector and the 99% port of the coupler. The group velocity dispersion at 1552 nm for the single mode fiber and the EDF are 18 and −16 ps/km/nm, respectively, and the laser cavity has a negative net dispersion.

### Preparation of the GSA

Graphene used in our experiment is commercial dispersive aqueous solution of graphene flakes with concentration of 0.48 wt% (Timesnano). The solution of graphene is diluted with 100 μL graphene dispersive water solution and 5 mL pure ethanol and sonicated for 5 min. After sonicating, the graphene dispersive solution is allowed to settle for several weeks. The graphene flakes are transferred onto the facet of fiber pigtail by optically driven deposition method. Optical radiation from a 1480 nm single mode fiber laser is injected into the fiber with the output power of ~13 dBm and propagates through the fiber while the fiber is dipped into the diluted graphene solution for 2 min.

### Designing of the RD-FBG

The RD-FBG array consists of 20 FBGs with the same central wavelength of 1552.5 nm, reflectivity about 4% and length of 3 mm for each FBG, which is fabricated in EDF through hydrogen loading and ultra-violet exposing technique. The separation distance between neighboring FBG is chosen in the range of 3~7 mm under uniform distribution, which are equal to 5.62 mm, 3.14 mm, 6.40 mm, 6.74 mm, 5.15 mm, 6.03 mm, 5.97 mm, 4.57 mm, 5.62 mm, 3.68 mm, 5.82 mm, 3.13 mm, 4.11 mm, 3.18 mm, 3.39 mm, 6.29 mm, 5.78 mm, 4.27 mm, 6.80 mm. The total length of the RD-FBG array is about 15 cm. An electric moving stage was used to precisely regulate the shift distance of the fiber while inscription the FBGs. The reflection/transmission spectrum of the FBG array is measured by a wavelength-swept-laser-based optical spectrum analyzer (OSA, Agilent with spectral resolution of 0.1 pm). The Agilent OSA consists of two parts, a tunable laser source (Agilent 81960 A, resolution of 0.1 pm at 1550 nm) and an optical power meter (Agilent N7744A). So it is based on a scanning light source for measuring the spectrum of passive devices.

### Simulation of frequency characteristics

Propagation of light wave in one dimensional medium is described by the transfer matrix, *M*_*i*_, which relates the amplitudes of the forward- (i.e., 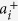) and backward-propagating (i.e., 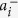) waves on the right side of each element to those on the left side^36^:


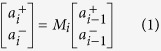


In the simulation, low reflectivity FBG is simplified into a point reflector with the same reflectivity, and the whole cavity is assumed to be a lossless and passive structure. Thus, *M*_*i*_ can be written as:


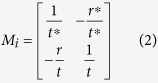


where *r/t* denotes reflection/transmission coefficient of the amplitude, and ‘*’stands for complex conjugate. The spacing distance between neighboring reflectors is described as transfer matrix *m*_*i*_:


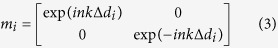


where *n* is effective refractive index, *k* is wave vector, Δ*d* is separation distance between neighboring reflectors. Then transfer matrix for the total structure is *M*:


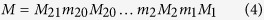


Considering the boundary condition 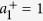, 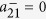, it is easy to obtain the reflection and transmission coefficient of the proposed structure:


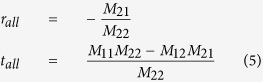


In this way, reflection and transmission spectra can be obtained (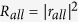 and 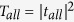). It is noted that in the simulation, the separation distance between neighboring point FBG reflector coincides with those in the RD-FBG array and the distance of the long cavity (point reflector-RDFBG array) also equals that in the experiment. The simulating precision shall be set to be specific values to respectively reflect the frequency information of Regime I and II. In addition, this numerical method is a steady-state analysis without considering the variation of time. So with specific separation distance values, reflection/transmission coefficient of the simplified point reflector and numerical resolution, specific reflection spectra can be obtained uniquely.

## Additional Information

**How to cite this article**: Ma, R. *et al*. Quasi mode-locking of coherent feedback random fiber laser. *Sci. Rep.* **6**, 39703; doi: 10.1038/srep39703 (2016).

**Publisher's note:** Springer Nature remains neutral with regard to jurisdictional claims in published maps and institutional affiliations.

## Supplementary Material

Supplementary Information
