# Supplementary material for: Quasi mode-locking of coherent feedback random fiber laser
Source: Sci Rep. 2016 Dec 22;6:39703. doi: 10.1038/srep39703 (PMC5177947; doi:10.1038/srep39703)
Supplement: Supplementary Information [file srep39703-s1.doc]

**Supplementary Information**

**Quasi mode-locking of coherent feedback random fiber laser**

R. Ma^1^, W. L. Zhang^1^*, X. P. Zeng^1^, Z. J. Yang^1^, Y. J. Rao^1^*, B. C. Yao^1^, C. B. Yu^1^, Y. Wu^1^ and S. F. Yu^2^

*^1^ Key Laboratory of Optical Fiber Sensing and Communications (Education Ministry of China), University of Electronic Science and Technology of China, Chengdu 610054, China*

*^2^ Department of Applied Physics, the Hong Kong Polytechnic University, Hung Hom, Hong Kong SAR, China*

^*^ Corresponding author: yjrao@uestc.edu.cn and wl_zhang@uestc.edu.cn

**I. Dynamical spatio-temporal characteristics of the Q-switched mode-locking regime**

Spatio-temporal dynamics of the Q-switched mode-locking regime was applied in our analysis to visualize the intensity evolution of the mode-locking sub-pulses in every Q-switched event. As is illustrated in the main manuscript the spatio-temporal map contains 10 cascaded Q-switched events without considering the interval separation time between neighboring Q-switched events. For each Q-switched event the intensity dynamics were consecutively divided apart into different segments with the period corresponding to low-frequency round trip time (~182 ns), then these segments were arranged in consecutive row to form the matrix of spatio-temporal map in two-dimensional graph, as is shown in Figure S1 a. In this way, the serial number of the row is represented by round trip number in the vertical axis.

Figure S1 b and d represent the temporal profiles of the selected sub-pulse in the fifth and eighth Q-switched events (red and purple dashed line in Figure S1a, respectively), while Figure S1 c and e give the undivided consecutive sub-pulse evolution of the same Q-switched events in Figure S1 b and d respectively. It is worth to note that the tilt of the intensity profile is cause by uncertainty in the round-trip time value. The period (182 ns) we used to divide the cascaded intensity sequence is always slightly different with the actual round-trip time value. So the intensity inevitably appear tilted with the accumulated error over many round trips. However it is clear that the intensity profiles for sub-pulses within one Q-switched pulse are parallel with each other, which reflects good quality of mode locking.

It is observed that shape of the temporal profile keeps unchanged in one Q-switched event, however, they are different in different Q-switched event. So the spatio-temporal map helps to visualize the sub-pulse temporal profile evolution in individual Q-switched event and compare the differences in different Q-switched events.


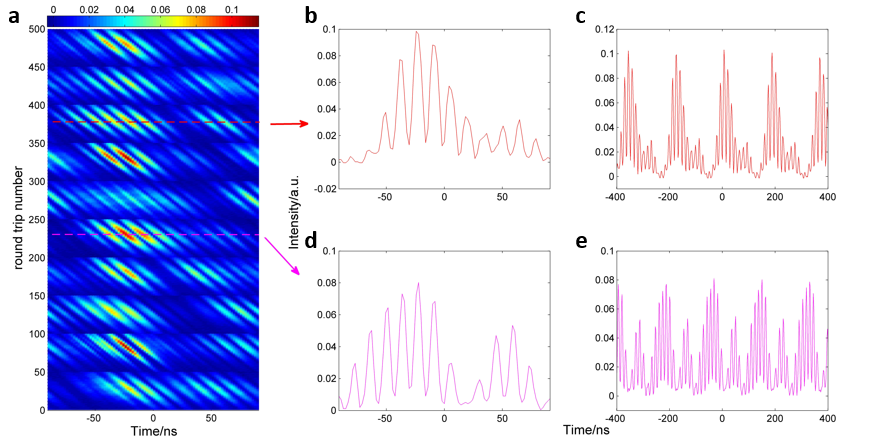


**Figure S1 | Principle of spatio-temporal dynamical map.** **a,** Spatio-temporal intensity dynamics of mode-locking regime for cascaded Q-switched events. **b** and **d** represent the sub-pulses of the fifth and eighth Q-switched events in **a** respectively. **c** and **e** represent the undivided consecutive sub-pulses of the fifth and eighth Q-switched events.

**II. Comparison of frequency characteristic in Fabry-Perot cavity and RD-FBG cavity**

To verify the function of the random distributed fiber Bragg grating (RD-FBG), we compare the radio frequency (RF) spectra of a Fabry-Perot cavity and the RD-FBG cavity.

First we use the same structure in out experiment except for replacing the RD-FBG array with a single narrow band fiber Bragg grating (FBG, reflectivity >90%, 3dB bandwidth 0.2 nm) to compose a conventional Fabry-Perot cavity. Q-switched pulse can be generated but no obvious mode locking event is observed, as is shown in Figure S2a and its inset. Figure S2b gives the RF spectrum of the temporal waveform (To make the data accurately reflecting the reality and eliminate accidental factors, we recorded 20 groups of the pulse series with each time scale of 4 ms every 30 seconds.). It is clear that in the single FBG formed cavity no high order frequency pass band and stop band exit in the RF spectrum as in the RD-FBG cavity.

Similarly, we recorded 20 groups of pulse series for the RD-FBG formed cavity (as in the main manuscript) and the RF spectrum is shown in Figure S3. It can be demonstrated that under the same circumstances the RD-FBG provides collective resonant oscillation between the localized modes and the global cavity modes, which is helpful for sub-pulses mode-locking with high order harmonic frequency.


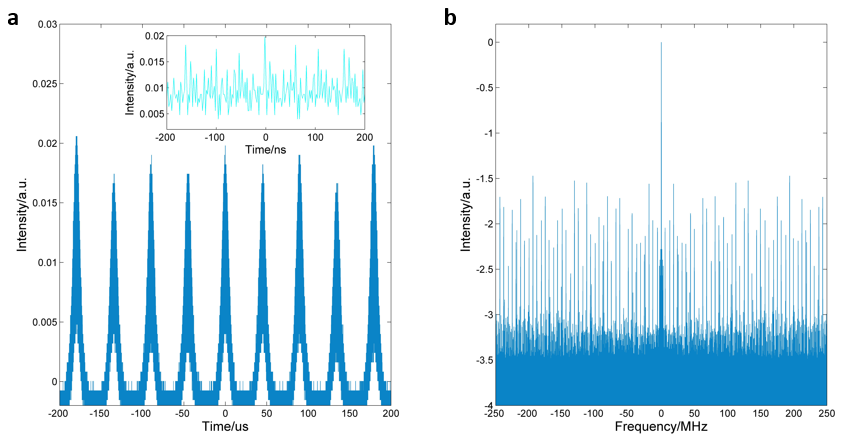


**Figure S2 | Temporal and frequency characteristics of single FBG formed Q-switched laser. a,** Oscilloscope trace of Q-switched trains under pump power of 59.61 mW. Inset: close-up view of sub-pulses within the Q-switched event. **b,** Radio frequency spectrum of the total 20 group pulse series with time scale of 4 ms for each group.


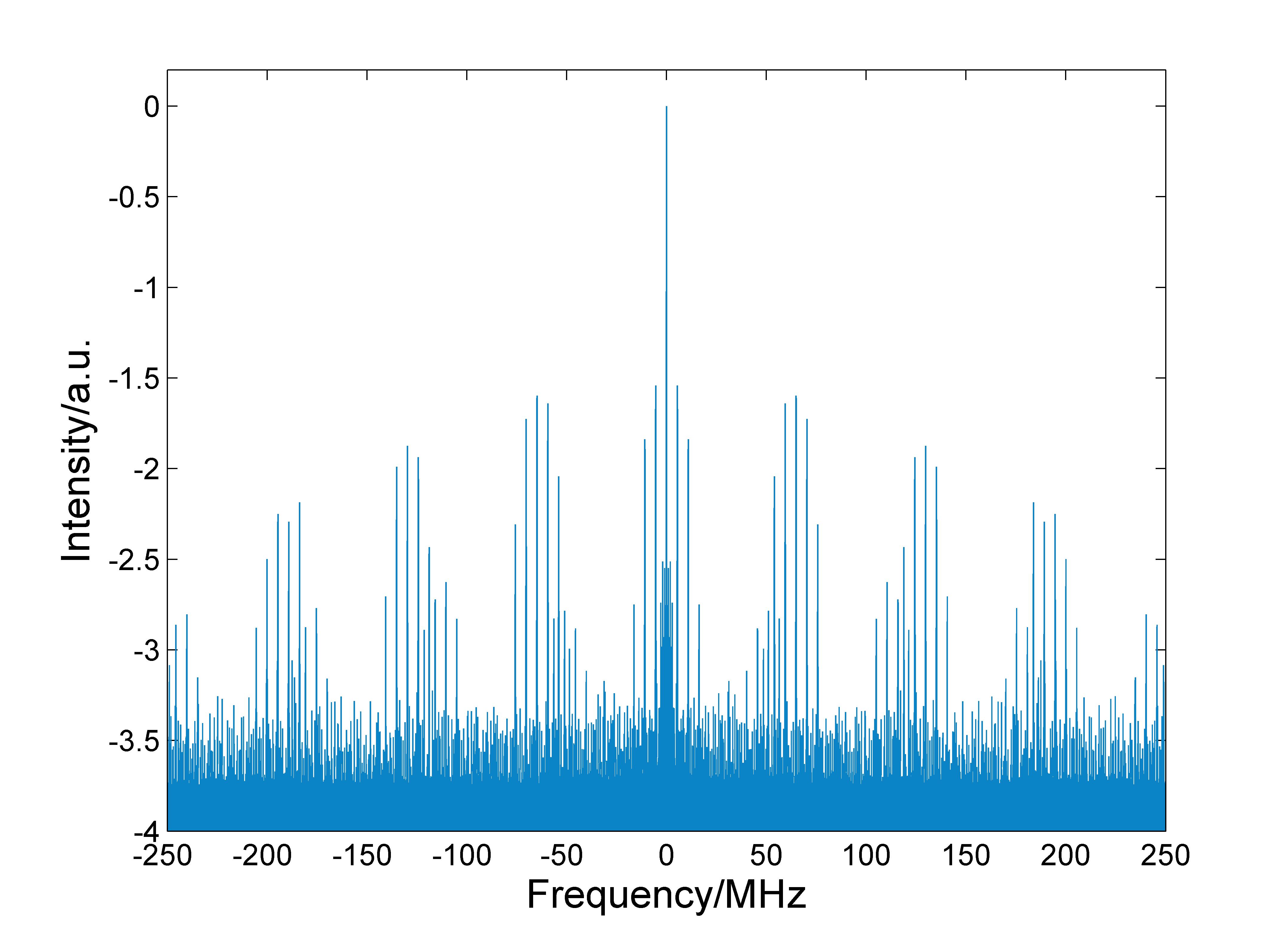


**Figure S3 | Temporal and frequency characteristics of RD-FBG formed Q-switched mode-locking laser.** Radio frequency spectrum of the total 20 group pulse series with time scale of 4 ms for each group.
